# Prevalence and Characteristics of Low-renin Hypertension in a Primary Care Population

**DOI:** 10.1210/jendso/bvae113

**Published:** 2024-06-05

**Authors:** Sonali S Shah, Renata Libianto, Stella May Gwini, Grant Rusell, Morag J Young, Peter J Fuller, Jun Yang

**Affiliations:** Centre for Endocrinology and Metabolism, Hudson Institute of Medical Research, Clayton, Victoria 3168, Australia; Department of Endocrinology, Monash Health, Clayton, Victoria 3168, Australia; Department of Molecular and Translational Science, Monash University, Clayton, Victoria 3168, Australia; Centre for Endocrinology and Metabolism, Hudson Institute of Medical Research, Clayton, Victoria 3168, Australia; Department of Endocrinology, Monash Health, Clayton, Victoria 3168, Australia; Centre for Endocrinology and Metabolism, Hudson Institute of Medical Research, Clayton, Victoria 3168, Australia; School of Public Health and Preventive Medicine, Monash University, Clayton, Victoria 3168, Australia; Department of General Practice, Monash University, Notting Hill, Victoria 3168, Australia; Centre for Endocrinology and Metabolism, Hudson Institute of Medical Research, Clayton, Victoria 3168, Australia; Baker Heart and Diabetes Institute, Prahran, Victoria 3004, Australia; Centre for Endocrinology and Metabolism, Hudson Institute of Medical Research, Clayton, Victoria 3168, Australia; Department of Endocrinology, Monash Health, Clayton, Victoria 3168, Australia; Department of Molecular and Translational Science, Monash University, Clayton, Victoria 3168, Australia; Centre for Endocrinology and Metabolism, Hudson Institute of Medical Research, Clayton, Victoria 3168, Australia; Department of Endocrinology, Monash Health, Clayton, Victoria 3168, Australia; Department of Molecular and Translational Science, Monash University, Clayton, Victoria 3168, Australia

**Keywords:** low-renin, hypertension, prevalence

## Abstract

**Introduction:**

Low-renin hypertension is an underrecognized subtype of hypertension with specific treatment options. This study aims to identify the prevalence in primary care and to compare patient characteristics to those with normal-renin hypertension and primary aldosteronism (PA).

**Methods:**

In a cohort study, patients with treatment-naïve hypertension were screened for PA with plasma aldosterone and direct renin concentrations. Patients with an elevated aldosterone-to-renin ratio [≥70 pmol/mU (≥2.5 ng/dL:mU/L)] underwent confirmatory testing. All screened patients were then classified as having (1) normal-renin hypertension, (2) low-renin hypertension (direct renin concentration <10mU/L (plasma renin activity ∼<1 ng/mL/hour) and not meeting the criteria for PA), or (3) confirmed PA.

**Results:**

Of the 261 patients, 69 (26.4%) had low-renin hypertension, 136 (51.9%) had normal renin hypertension, and 47 (18.0%) had PA. Patients with low-renin hypertension were older and more likely to be female compared to normal-renin hypertension (57.1 ± 12.8 years vs 51.8 ± 14.0 years, *P* < .05 and 68.1% vs 49.3%, *P* < .05, respectively) but similar to PA (53.5 ± 11.5 years and 55.3%). However, in an adjusted binomial logistic regression, there was no association between increasing age or sex and low-renin hypertension. The median aldosterone concentration was lower compared to patients with normal-renin hypertension and PA: 279 pmol/L (216-355) vs 320 pmol/L (231-472), *P* < .05 and 419 pmol/L (360-530), *P* < .001.

**Conclusion:**

At least a quarter of treatment-naïve hypertensive patients in primary care had a low direct renin concentration but did not meet the criteria for PA. Patient characteristics were similar, aside from a lower aldosterone concentration compared to patients with normal-renin hypertension and PA. Further research is needed to understand the underlying pathophysiology of low-renin hypertension and the optimal first-line treatment.

Low-renin hypertension was identified as a subtype of hypertension in the 1960s when plasma renin activity assays first became available [1]. Though somewhat arbitrary, a plasma renin activity <1 ng/mL/hour is commonly accepted as the definition of “low-renin” [1]. It is hypothesized that the low levels of renin in the context of hypertension are due to negative feedback from expanded extracellular fluid volume, excess salt reabsorption and/or mineralocorticoid receptor activation. As such the renin measurement provides insight into the underlying pathophysiology in an individual with hypertension and may guide the choice of optimal anti-hypertensive medication [2, 3]. A recent meta-analysis found that mineralocorticoid receptor antagonists were superior in lowering systolic blood pressure in low-renin hypertension compared to the commonly prescribed first-line class of antihypertensives, angiotensin-converting enzyme inhibitors, and angiotensin receptor blockers, with a mean difference of −6.8 mmHg [4]. Other studies have demonstrated better blood pressure-lowering with diuretics and epithelial sodium channel inhibitors in people with low-renin hypertension compared to beta-blockers and angiotensin receptor blockers [5, 6]. However, translation to clinical practice has been limited by the availability of renin measurements in primary care and common factors, such as antihypertensive medications, make interpretation of renin measurements more challenging [7-11]. In addition, evidence supporting the superiority of mineralocorticoid receptor antagonists and diuretics in lowering blood pressure in low-renin hypertension is inconsistent and dated [12].

Over the years, analytical methods for measuring renin have evolved; the direct renin concentration immunoassay is accessible with a quick turnaround time at most major pathology laboratories and thus has largely superseded plasma renin activity assays [11]. Furthermore, with increased awareness of the prevalence and complications of primary aldosteronism (PA), a disease characterized by autonomous aldosterone production by 1 or both adrenal glands, renin, aldosterone concentrations, and aldosterone-to-renin ratios (ARR) are now more commonly ordered in efforts to increase detection [11, 13, 14]. Interest in low-renin hypertension has thus been revived as clinicians are now presented with the challenge of managing patients with low-renin hypertension who do not meet the criteria for PA. Previous studies report that 37% to 57% of patients with “essential hypertension” have low plasma renin activity [15, 16]. However, this may not be generalizable to primary care as these figures are derived from interventional trials at specialist centers with small sample sizes. To date, there are no prevalence studies for low-renin hypertension in primary care, where hypertension is most commonly managed [17]. Therefore, the objective of this study was to identify the prevalence of low-renin hypertension in an unselected primary care population to elucidate what proportion of the hypertensive population may benefit from personalized first-line treatment and to identify any distinguishing clinical and biochemical characteristics compared to those with normal renin hypertension or PA.

## Methods

### Study Design and Patient Eligibility

We conducted a retrospective analysis of data collected in a prospective PA prevalence study in primary care in Victoria, Australia [18]. Between 2017 and 2021, treatment-naïve adult patients diagnosed with hypertension, defined as blood pressure ≥140/90 mmHg on 3 measurements at the general practice clinic, were consented and enrolled in the PA prevalence study [18]. The current study is an extension of the published PA prevalence study with an additional 12 months of enrolment.

### Measurements

Patients were referred by their general practitioners for a PA screening blood test, the ARR, along with serum electrolytes and renal function. The ARR was derived from the plasma aldosterone concentration and the direct renin concentration. These were measured using chemiluminescent immunoassays (LIAISON, DiaSorin Cat#310450, RPID: AB_2889867 and Cat#310470, RPID: AB_2889866, respectively) at the following Victorian pathology laboratories: Monash Pathology, Melbourne Pathology, Australian Clinical Labs, Dorevitch Pathology, 4Cyte Pathology, Austin Pathology, and St. Vincent's Pathology. For the direct renin concentration assay, the within-run coefficients of variation were 6.6% at 24.0 mU/L and 1.4% at 92.4 mU/L; total coefficients of variation were 10.0% at 24.0 mU/L and 4.5% at 92.4 mU/L. For the aldosterone assay, the within-run coefficients of variation were 3.5% at 188 pmol/L (6.8 ng/dL) and 1.8% at 798 pmol/L (28 ng/dL); total coefficients of variation were 9.6% at 188 pmol/L (6.8 ng/dL) and 5.6% at 798 pmol/L(28 ng/dL).

### Classification of Normal Renin Hypertension, Low-renin Hypertension, and PA

PA was diagnosed in line with clinical practice guidelines [11]. Patients with a positive screening test, defined as an ARR greater than or equal to 70 pmol/mU (≥2.5 ng/dL:mU/L), were referred to the Monash Health Endocrine Hypertension Clinic for further investigation of PA. If the elevated ARR was confirmed on repeat testing, patients underwent a saline suppression test to confirm PA diagnosis, defined as a postsaline suppression test plasma aldosterone concentration >140 pmol/L (5.1 ng/dL) supine or >170 pmol/L (6.1 ng/dL) seated. If the repeat ARR was normal [<70 pmol/mU (<2.5 ng/dL:mU/L)], patients continued their care with their general practitioners. Results for the prevalence of PA and subtyping of PA among patients seen between 2017 and 2020 have been reported by Libianto et al [18]. For patients who did not have PA, a threshold of direct renin concentration <10mU/L (approximately equates to plasma renin activity <1 ng/mL/hour) was used to define low-renin hypertension [11]. The third group of patients were those who did not have PA and had direct renin concentration ≥10 mU/L (ie, normal renin hypertension group).

### Clinical and Biochemical Data

The following data were collected at the time of screening: age, sex, systolic and diastolic blood pressure, body mass index (weight in kilograms divided by square of height in meters), fasting serum lipids, and plasma glucose levels. General practices were contacted in 2023 to obtain missing data corresponding to the time of the screening plasma aldosterone concentration and direct renin concentration measurements.

### Statistical Analysis

Mean and standard deviation (SD) were used to describe normally distributed continuous data, and median, 25th, and 75th percentiles were used to describe nonnormally distributed data. Frequencies of categorical data were reported as percentages. One-way ANOVA was used to compare the 3 subgroups for variables that were normally distributed data. Where the ANOVA null hypothesis was rejected, pairwise comparison was done using Bonferonni or Games–Howell depending on homogeneity of variance. The Kruskal–Wallis H test was used for the subgroup comparisons of nonnormally distributed data, and Chi-square was used to compare categorical variables. The prevalence of low-renin hypertension was reported as a percentage with a 95% confidence interval (CI) obtained using bootstrapping for 1000 samples. After removing patients diagnosed with PA, a binomial logistic regression analysis was undertaken to explore the relationship between patient characteristics, biochemistry, comorbidities, and renin subgroup. The significance threshold was set as a 2-sided *P*-value <.05. IBM SPSS v28 was used for statistical analysis.

### Ethics Approval

The study was approved by the Monash Health Human Research Ethics Committee (HREC/16/MonH/390).

### Reporting

Findings are reported in line with the Strengthening the Reporting of Observational studies in Epidemiology framework [19].

## Results

Of the 80 general practice clinics invited to participate in this study, 31 were agreeable. The majority of the practices were located in metropolitan Melbourne: 29 out of 31. Over the 4 years, data was extracted from the 261 patients who met the eligibility criteria.

### PA Screening

**Figure 1.**
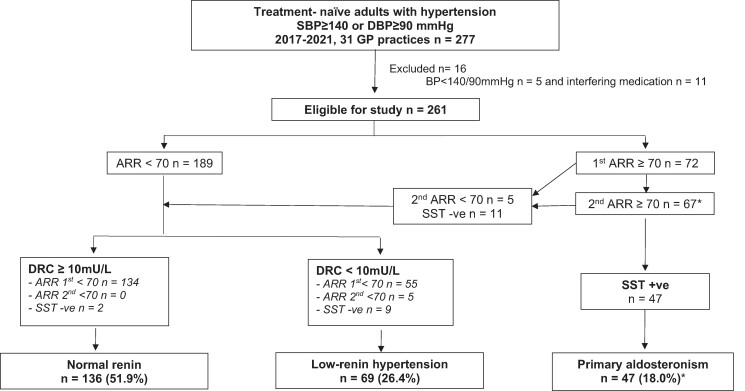
Prevalence of low-renin hypertension. *n = 9 did not have saline suppression test and were classified as having “possible primary aldosteronism” instead.

Abbreviations: ARR, aldosterone-renin ratio; DBP, diastolic blood pressure; DRC, direct renin concentration; GP, general practitioner; n: number of patients; PAC, plasma aldosterone concentration; SBP, systolic blood pressure; SST, saline suppression test, +ve, positive, −ve, negative.

Out of the 261 patients, 72 had a positive screening result for PA [ARR ≥70 pmol/mU (≥2.5 ng/dL:mU/L)]; 9 patients declined or were unable to undergo a saline suppression test and were categorized as having “possible PA” (Fig. 1). These patients were included in the analysis for prevalence but not in the between-group comparisons. Fifty-eight patients underwent further investigation with a saline suppression test; 47 were confirmed as having PA and the rest were reclassified as low-renin hypertension (n = 9) and normal renin hypertension (n = 2) based on their initial direct renin concentration level. Confirmatory testing was not bypassed as no patient had spontaneous hypokalemia, plasma renin below detection levels, and a plasma aldosterone concentration >550 pmol/L (20 ng/dL). Out of the 261 patients,189 patients had an ARR < 70 and were classified as having normal renin hypertension (n = 134) or low-renin hypertension (n = 55) (Fig. 1).

### Prevalence of Low-renin Hypertension

The prevalence of low-renin hypertension was 26.4% (n = 69, 95% CI 21.5-31.8) (Fig. 1). Of the 69 patients who had a direct renin concentration <10 mU/L, 55 (80%) had an initial ARR <70 pmol/mU (<2.5 ng/dL:mU/L), 5 (7%) had an initial ARR ≥70 pmol/mU (≥2.5 ng/dL:mU/L) but <70 pmol/mU (<2.5 ng/dL:mU/L) on repeat testing, and 9 (13%) had 2 ARR ≥70 pmol/mU (≥2.5 ng/dL:mU/L) but had adequate suppression of aldosterone postsaline infusion.

### Patient Demographics

Compared to the normal renin hypertension group, those with low-renin hypertension had more female patients (68.1% vs 49.3%, *P* = .031) and were older (57.1 ± 12.8 years vs 51.8 ± 14.0 years *P* = .022) (Table 1). There was no difference in age and proportion of females compared to PA: 53.5 ± 11.5 years and 55.3%, respectively.

**Table 1.** Patient characteristics

	Normal-renin hypertension n = 136	Low-renin hypertension n = 69	Primary aldosteronism n = 47	Comparison between groups (overall *P*-value)
Age mean ± SD (years)	51.8 ± 14.0^*^	57.1 ± 12.8	53.5 ± 11.5	.030
Sex % female (number of patients)	49.3 (67)^*^	68.1 (47)	55.3 (26)	.030
Systolic blood pressure mean ± SD (mmHg)	157 ± 16	158 ± 16	156 ± 17	.818
Diastolic blood pressure mean ± SD (mmHg)	95 ± 11	93 ± 12	94 ± 12	.522
Body mass index mean ± SD (18.5-24.9 kg/m^2^)	29.7 ± 5.5	29.5 ± 5.3	28.7 ± 3.9	.601
Plasma aldosterone median (25th, 75th centile) (70-1090 pmol/L (2.5-39.3 ng/dL)	320 (231, 472)^*^	279 (216, 355)	419 (360, 530)^†^	<.001
Aldosterone-renin ratio median (25th, 75th centile) (<70 pmol/mU (<2.5 ng/dL:mU/L))	16 (9, 27)^†^	47 (34, 61)	106 (84, 140)^†^	<.001
Serum sodium mean ± SD (135-145 mmol/L)	141 ± 2	141 ± 2	141 ± 2	.255
Serum potassium mean ± SD (3.5-5.2 mmol/L)	4.37 ± 0.35	4.44 ± 0.36	4.26 ± 0.30^†^	.029
Serum bicarbonate mean ± SD (22-32 mmol/L)	28 ± 2	27 ± 3	28 ± 3	.525
Serum creatinine mean ± SD (M: 60-110, F: 45-90 umol/L)	75 ± 16	71 ± 16	73 ± 13	.340
eGFR mean ± SD (>90 mL/min/1.73 m^2^)	93 ± 15	88 ± 17	91 ± 12	.157
Fasting plasma glucose mean ± SD (4.0-6.0 mmol/L)	5.5 ± 0.7	5.6 ± 1.3	5.3 ± 0.5	.251
Total cholesterol mean ± SD (<5.5 mmol/L)	5.3 ± 1.0	5.3 ± 1.0	5.3 ± 0.9	.952
High-density lipoprotein mean ± SD (M: > 1.0, F: > 1.2 mmol/L)	1.4 ± 0.4	1.6 ± 0.5	1.5 ± 0.5	.120
Low-density lipoprotein mean ± SD (<3.0 mmol/L)	3.2 ± 0.9	3.1 ± 0.8	3.2 ± 0.8	.775
Triglyceride mean ± SD (<2.0 mmol/L)	1.6 ± 1.1	1.4 ± 0.8	1.3 ± 0.6	.146

Thirteen out of 136 participants classified as “normal-renin hypertension” had a direct renin concentration above the reference range (≥46 mU/L).

The reference range for normal values is shown in parentheses. Aldosterone 1 pmol/L = 0.04 ng/dL.

Missing data, systolic and diastolic blood pressure: NRH (n = 14), LRH (n = 2); body mass index: NRH (n = 40), LRH (n = 15), PA (n = 3); sodium: NRH (n = 1), LRH (n = 1), PA (n = 1); potassium and creatinine, eGFR: NRH (n = 1), LRH (n = 1); bicarbonate: NRH (n = 5), LRH (n = 2), PA (n = 1); fasting plasma glucose: NRH (n = 26), LRH (n = 12), PA (n = 7); total cholesterol: NRH (n = 10), LRH (n = 3), PA (n = 2); high-density lipoprotein: NRH (n = 13), LRH (n = 3), PA (n = 4); low-density lipoprotein: NRH (n = 14), LRH (n = 5), PA (n = 4); triglyceride: NRH (n = 10), LRH (n = 3), PA (n = 3).

Abbreviations: eGFR, estimated glomerular filtration rate calculated according to the Chronic Kidney Disease Improved Prediction Equations method; F, female; LRH, low-renin hypertension; M, male; NRH, normal renin hypertension; PA, primary aldosteronism.

^*^ *P* < .05 and ^†^*P* < .01 compared to low-renin hypertension group.

### Plasma Aldosterone Concentration, Direct Renin Concentration, and General Biochemistry

**Figure 2.**
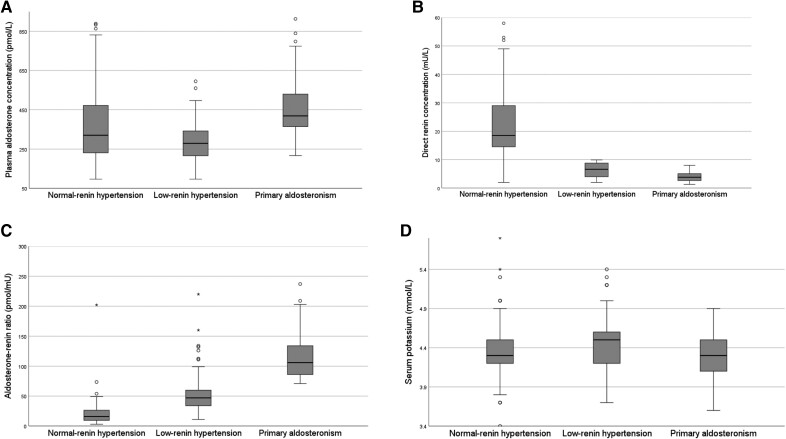
(A) Plasma aldosterone concentration, (B) direct renin concentration, (C) aldosterone-to-renin ratio, and (D) serum potassium in each subgroup (initial blood test) in each group.

The median plasma aldosterone concentration in the low-renin hypertension group was 279 pmol/L (216-355). This was lower compared to the plasma aldosterone concentration in both the normal renin hypertension group, 320 pmol/L (231-472), *P* = .015, and the PA group, 419 pmol/L (360-530), *P* < .001 (Fig. 2A), whereas the median direct renin concentration was lower in low-renin hypertension, 6.6 mU/L (4.0-8.9), compared to normal renin hypertension, 18.5 mU/L (14.5-29.0), but higher compared to PA, 3.8 mU/L (2.6-5.1), *P* < .001 (Fig. 2B). This resulted in a higher median [ARR in the low-renin hypertension group: 47 pmol/mU (34-61)] compared to the normal renin hypertension group, 16 pmol/mU (9-27), but lower ARR compared to the PA group, 106 pmol/mU (84-140), *P* < .001 (Fig. 2C).

Serum potassium values were slightly higher in the low-renin hypertension group (4.44 ± 0.36 mmol/L) compared to patients with PA (4.26 ± 0.30 mmol/L) with a mean difference of 0.18 mmol/L (95% CI 0.02-0.33 *P* < .026) (Fig. 2D). Serum sodium and bicarbonate levels and estimated glomerular filtration rates were similar across all groups (Table 1).

### Blood Pressure, Body Mass Index and Metabolic Profile

The mean systolic blood pressure in the low-renin hypertension group was 158 ± 16 mmHg and the diastolic blood pressure was 93 ± 12 mmHg. The mean body mass index was in the overweight range at 29.5 ± 5.3 kg/m^2^, mean fasting plasma glucose was 5.6 ± 1.3 mmol/L, and total cholesterol was 5.3 ± 1.0 mmol/L. These were also similar across all groups (Table 1).

### Relationship Between Patient Characteristics and Low-renin Hypertension

After removing patients diagnosed with PA, a binomial logistic regression model was run to assess the effect of patient clinical characteristics, biochemical parameters, and comorbidities on having low-renin hypertension. In the unadjusted analyses, age, sex, and high-density lipoprotein were associated with low-renin hypertension (Table 2), such that, for every 1-year increase in age, the odds of having low-renin hypertension increased by 3% while female sex and a 1 mmol/L increase in high-density lipoprotein increased the odds by 2-fold, compared with normal renin hypertension. (Table 2) There was a trend toward an association between decreasing estimated glomerular filtration rate (CKD-EPI) and having low-renin hypertension (odds ratio = 0.98, 95% CI 0.96-1.00, *P* = .074). When adjusted for other variables, there was a trend toward an association between female sex and having low-renin hypertension (odds ratio = 1.97, 95% CI 0.99-3.89, *P* = .053). However, age and high-density lipoprotein were no longer significantly associated with having low-renin hypertension (Table 2).

**Table 2.** Binomial logistic regression analysis of low-renin hypertension (reference group is normal renin hypertension)

	Unadjusted model			Adjusted model (for age, sex, eGFR and high-density lipoprotein)		
	Odds ratio	95% CI	*P*-value	Odds ratio	95% CI	*P*-value
Age	1.03	1.01-1.05	.010	1.02	0.99-1.05	.189
Sex (female)	2.20	1.20-4.04	.011	1.97	0.99-3.89	.053
Systolic blood pressure	1.00	0.99-1.02	.673	0.99	0.97-1.01	.510
Diastolic blood pressure	0.99	0.96-1.01	.279	0.99	0.96-1.02	.596
Body mass index	0.99	0.93-1.06	.809	0.98	0.91-1.05	.499
Serum sodium	1.09	0.95-1.25	.199	1.06	0.92-1.23	.416
Serum potassium	1.71	0.75-3.92	.202	1.46	0.60-3.55	.404
Serum bicarbonate	0.94	0.82-1.08	.415	0.93	0.80-1.08	.355
eGFR	0.98	0.96-1.00	.074	0.99	0.96-1.02	.439
Fasting plasma glucose	1.14	0.82-1.59	.434	1.06	0.73-1.54	.754
Total cholesterol	0.94	0.68-1.28	.674	0.87*^a^*	0.63-1.20	.402
High-density lipoprotein	2.01	1.01-3.98	.046	1.45	0.69-3.03	.326
Low-density lipoprotein	0.86	0.60-1.23	.402	0.89*^a^*	0.62-1.29	.544
Triglyceride	0.76	0.50-1.14	.180	0.73*^a^*	0.46-1.16	.182

Abbreviations: CI, confidence interval; eGFR, estimated glomerular filtration rate calculated according to the Chronic Kidney Disease Improved Prediction Equations method.

^*a*^Parameters were not adjusted for high-density lipoprotein in the adjusted model due to possible collinearity.

Systolic and diastolic blood pressure, body mass index, serum sodium, serum potassium, fasting plasma glucose, total cholesterol, low-density lipoprotein, and triglyceride were not associated with having low-renin hypertension, based on both the unadjusted and adjusted analysis for age, sex, estimated glomerular filtration rate, and high-density lipoprotein (Table 2).

## Discussion

There is limited data on the proportion of primary care patients who have hypertension with low renin despite evidence supporting the role of targeted antihypertensive therapy that differs from standard care for this type of hypertension [4]. The objective of this study was to identify the prevalence of low-renin hypertension in hypertensive people who do not meet current screening or diagnostic criteria for PA in primary care and whether there are any distinguishable clinical or biochemical characteristics compared to those patients with PA and normal renin hypertension. We found that 26.4% of treatment-naïve, primary care patients with blood pressure ≥140/90 mmHg met the criteria for low-renin hypertension. These patients were older and more likely to be female than those with normal renin hypertension and had a lower plasma aldosterone concentration than those with normal renin hypertension or PA. This is the first Australian study to report the prevalence of low-renin hypertension in primary care. Importantly, the prevalence we report is similar to recent studies done at specialist centers in China and India, which reported a prevalence of 26% (332 out of 1267 patients) and 32% (287 out of 896 patients), respectively [20, 21]. This represents a substantial proportion of people with hypertension and presents an opportunity to explore personalized treatment based on the underlying pathophysiology of the disease to improve long-term control of hypertension. At present, up to 2 out of 3 people with hypertension are considered to have uncontrolled hypertension [22]. This may be partly attributed to significant interindividual variability in the underlying mechanisms that are responsible for hypertension and thus the individual response to different classes of antihypertensive agents [23]. A recent systematic review and meta-analysis found that people with low-renin hypertension have a greater systolic blood pressure lowering effect with mineralocorticoid receptor antagonists compared to commonly used first-line antihypertensive class, angiotensin-converting enzyme inhibitors and angiotensin receptor blockers [4]. In particular, Weinberger et al found that in participants with low-renin hypertension, 8 weeks of monotherapy with eplerenone (100-200 mg/day), a mineralocorticoid receptor antagonist, lowered systolic blood pressure by 17 mmHg compared to 5 mmHg with losartan (50-100 mg/day) in those with direct renin concentration <9.4mU/L [24]. Baseline renin concentration has also been found to be a predictor of response to mineralocorticoid receptor antagonist in resistant hypertension in the PATHWAY-2 study, a multicenter, double-blind, crossover trial comparing spironolactone (25-50 mg), bisoprolol (5-10 mg), doxazosin modified release (4-8 mg), and placebo [25]. A recent open-label study found that following a renin-guided treatment approach reduced the total number of medications required to achieve a target blood pressure <140/90 mmHg by swapping existing antihypertensives for diuretics and calcium channel blockers in participants with a plasma renin activity ≤0.5 ng/mL/hour (∼direct renin concentration of 4-6 mU/L) [21]. Given that two-thirds of people with hypertension require 2 or more antihypertensives to achieve adequate blood pressure control and that compliance is inversely related to the number of medications prescribed, a personalized treatment method based on a now readily available biomarker is certainly attractive compared with the current add-on treatment strategy [26, 27]. An estimate of the cost-effectiveness of this strategy found that it was cost-neutral, if not cheaper, in younger patients or those at high risk of cardiovascular events [28]. Other advantages that were not addressed in this model include (1) possible lower lifetime medication cost and decreased side effects due to exposure to fewer medications and (2) increased detection of PA, a condition that has clear treatment options effective in blood pressure control and reducing cardiovascular risk independently of blood pressure [11].

Our data showed that patients with low-renin hypertension had a lower plasma aldosterone concentration compared to patients with normal renin hypertension and PA. Known causes of low-renin hypertension with low-normal aldosterone levels include diseases such as apparent mineralocorticoid excess, Liddle syndrome, Gordon syndrome, and congenital adrenal hyperplasia [29-32]. However, these diseases are rare and usually present at a young age (<20 years). Therefore, it is unlikely that the patients with low-renin hypertension in this study with a mean age of 57 years would remain treatment-naïve with these conditions. The majority of people with low-renin hypertension are undefined in terms of their underlying pathology and may encompass a less severe phenotype of the known causes of low-renin hypertension, ie, nonclassical apparent mineralocorticoid excess with partial loss of 11-beta-hydroxysteroid dehydrogenase type 2 enzyme activity and abnormalities in the genes involved in sodium reabsorption in the distal nephrons of kidneys [33-36]. Recent studies have demonstrated that autonomous aldosterone production occurs on a continuum and may be present in people with normal blood pressure and lower aldosterone concentration [37, 38]. Current screening and diagnostic thresholds for PA may thus miss the less severe end of the spectrum of renin-independent aldosteronism and be misclassified as having low-renin hypertension.

Unsurprisingly, patients with PA had a lower potassium concentration compared to those with low-renin hypertension, likely reflecting a higher degree of mineralocorticoid receptor activation and kaliuresis. Interestingly, blood pressure was similar between patients with PA and low-renin hypertension. This may be because patients with PA were diagnosed earlier than occurs in routine clinical practice in this study given the eligibility criteria of treatment-naïve patients with blood pressure ≥ 140/90 mmHg [11].

Patients with low-renin hypertension were on average 5 years older than those with normal renin hypertension. An inverse relationship between age and renin has been previously reported in many studies [39, 40]. Yin et al measured plasma renin activity in 274 patients with essential hypertension; the median plasma renin activity for 20 to 29-year-olds was 5.6 ng/mL/hour compared to 2.3 ng/mL/hour in those aged 60 years or older [39]. Interestingly, a similar but less pronounced difference in plasma renin activity was seen in the 153 healthy control subjects: 4.1 ng/mL/hour vs 2.5 ng/mL/hour. Possible mechanisms underlying the decrease in renin with age include reduced renal mass and/or 11-beta-hydroxysteroid dehydrogenase 2 enzyme function, the appearance of aldosterone-producing adrenal cell clusters with age, and/or impaired responsiveness to baroceptor-and renal beta1-adrenergic receptor stimulation [41-43]. This raises the question of whether it would be more appropriate to have age-specific reference ranges for renin and thresholds for the classification of low-renin hypertension.

We also found a female predominance in the low-renin hypertension group compared to normal renin hypertension. This has been noted in previous randomized controlled trials with 62% to 79% of the low-renin hypertension population being female (mean age 44-47 years) [15, 16]. However, this has not been a consistent finding. A female predominance was not found by Luo et al in a recent observational study in China (46% female; mean age of participants 47 years) or in randomized controlled studies reported by Vaughan et al (49% female; mean age 55 years) and Solheim et al (49% female; mean age 59 years) [20, 44, 45]. A possible explanation is the effect of sex hormones on renin concentration. In our study, 95% of women with low-renin hypertension were between the ages of 53 and 61 years and presumed to be postmenopausal. The use of oral or transdermal estrogen replacement therapy has been reported to lower direct renin concentration (not plasma renin activity) in postmenopausal women due to estrogen-mediated reduction in sympathetic activity [46]. In a cross-sectional study of healthy middle-aged participants, Schunkert et al found that the mean direct renin concentration in 107 postmenopausal women on estrogen replacement therapy was 12.2 mU/L compared to 16.6 mU/L in postmenopausal women not on estrogen replacement therapy and 20.5 mU/L in 342 men, *P* < .01. This difference was noted irrespective of estrogen formulation or the mode of delivery (oral or transdermal). We were unable to perform a subgroup analysis to explore the impact of estrogen replacement as this information was not available. However, an Australian cross-sectional study reported that only 13% of women use menopausal hormone replacement therapy and therefore the use of estrogen replacement therapy would have had a limited effect on the renin concentration in our study [47].

Strengths of this study include that this was a primary care study in a culturally and linguistically diverse population in Victoria, Australia. As such, it is generalizable to the Australian population with hypertension. Recruiting from primary care facilitated the enrolment of treatment-naïve hypertensive patients, thereby removing the effect of antihypertensives on renin and aldosterone measurements. Furthermore, patients who were suspected of having PA had access to a specialist center and were offered confirmatory testing to ensure an accurate distinction between PA vs low-renin hypertension.

Limitations of the study include the generalizability to other populations including rural populations or younger people with hypertension. In addition, we cannot be sure that all primary care patients meeting the eligibility criteria were screened; ie, we are not able to exclude any potential selection bias towards screening those at higher risk of having PA. In addition, some patients may have been misclassified as most patients with low-renin hypertension or normal renin hypertension were categorized based on single aldosterone and renin measurements, both of which can be affected by various physiological stimuli [11]. However, the effect of this on our data is likely to be small as standardized instructions on timing (2 hours after waking) and posture (blood sample drawn in a seated position) were provided. Furthermore, diagnostic cutoffs make disease states binary. This is likely a simplistic interpretation of clinical test outcomes. Most diseases are a continuum, and as such a direct renin concentration threshold of <10mU/L and ARR screening cutoff of 70 pmol/mU (2.5 ng/dL:mU/L) for PA may miss some patients with low-renin hypertension or PA. Using a lower ARR threshold of 50 pmol/mU (1.8 ng/dL:mU/L) as a positive screening test for PA, as done in some centers, 35% (n = 92) instead of 28% (n = 72) of patients would have been invited to have confirmatory testing for PA. As such, our estimates of the proportion of primary care hypertensive patients with PA may be conservative. Other data that would have been interesting to further characterize the low-renin hypertension group but are not available in this study include ethnicity and 24-hour urine sodium excretion, a surrogate measure of salt intake [48]. Lastly, given that this was a retrospective analysis of data collected for the PA prevalence study, a sample size calculation was not performed [18]. To increase the precision of the estimated prevalence of low-renin hypertension in primary care and for subgroup analysis based on sex, age groups, and ethnicity, future prospective prevalence studies with a larger sample size are needed.

In conclusion, this study highlights that a large proportion of people with hypertension in primary care have low renin but do not meet the current screening and diagnostic criteria for PA. Characteristics of people with low-renin hypertension are similar to those with normal renin hypertension and PA, aside from a lower plasma aldosterone concentration, older age, and slight female predominance. As renin measurements become more accessible in routine clinical care, further research is warranted to explore the role of renin-guided personalized antihypertensive treatment in primary care with the potential to benefit a large number of people. Such a strategy may lead to reduced medication burden and therefore improved compliance, better blood pressure control, and reduced long-term sequelae of hypertension.

## Data Availability

Some or all datasets generated during and/or analyzed during the current study are not publicly available but are available from the corresponding author upon reasonable request.

## Abbreviations

ARR: aldosterone-renin ratio
CI: confidence interval
PA: primary aldosteronism
